# Right Internal Mammary Artery Perforation and Large Mediastinal Hematoma Formation as a Complication of Radial Access Coronary Angiography: A Case Report

**DOI:** 10.1002/ccr3.71533

**Published:** 2025-11-28

**Authors:** Seyed Alireza Mirhosseini, Mahsa Borjzadehgashtaseb, Mohammad Mohammadi, Soorena Khorshidi, Foad Amanollahi, Alisina Mirzaei, Armin Attar

**Affiliations:** ^1^ Department of Cardiovascular Medicine Shiraz University of Medical Sciences Shiraz Iran; ^2^ Student's Research Committee Shiraz University of Medical Sciences Shiraz Iran; ^3^ Department of Cardiovascular Medicine Jundishapour University of Medical Sciences Ahvaz Iran; ^4^ Department of Cardiovascular Medicine Kerman University of Medical Sciences Kerman Iran

**Keywords:** hemothorax, right internal mammary artery, trans radial angiography, vascular complication

## Abstract

Perforation of the right internal mammary artery (RIMA) is an extremely rare vascular complication associated with the transradial approach (TRA) to coronary angiography. A 75‐year‐old woman presented with weakness. ECG revealed atrial fibrillation with long pauses. Echocardiography showed severe left ventricular (LV) systolic dysfunction (LVEF: 20%). During coronary angiography, an unintended passage of a hydrophilic wire into the RIMA caused tissue staining indicative of severe spasm without perforation. The patient was initially managed conservatively, but then she experienced a significant drop in hemoglobin. Subsequent CT angiography revealed a mediastinal hematoma. The patient was emergently transferred to the catheterization laboratory, where angiography confirmed RIMA perforation with dye extravasation. Despite the unsuccessful balloon tamponade, a 2.5 × 21 mm covered stent was deployed, successfully sealing the perforation. This case is distinctive as one of the very few reported RIMA perforations complicating TRA coronary angiography. It emphasizes the importance of vigilance even when early angiography shows no clear perforation and highlights covered stenting as a life‐saving option when balloon tamponade is unsuccessful. Knowing the possible complications of the TRA, specifically during hydrophilic guidewire maneuvers, empowers us to recognize and manage these complications promptly.

## Graphical abstract

Representing the summary of findings and intervention in the case.

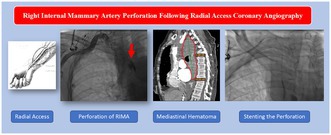

**Key Clinical Message**

Hydrophilic wires may become very troublesome during angiographic procedures and should not be used routinely.

**Abbreviations**
ACS: acute coronary syndrome
AF: atrial fibrillation
CAG: coronary angiography
CCU: cardiac care unit
CRT: cardiac resynchronization therapy
ECG: electrocardiogram
EDV: end‐diastolic volume
ESV: end‐systolic volume
GI: gastrointestinal
LV: left ventricle
LVEF: left ventricular ejection fraction
MPI: myocardial perfusion imaging
PCI: percutaneous coronary intervention
RIMA: right internal mammary artery
RV: right ventricle
TRA: transradial approach

## 1 Introduction

The transradial approach (TRA) has emerged as a favored alternative to the transfemoral approach for coronary angiography (CAG) and percutaneous coronary intervention (PCI). This preference stems from its association with reduced access site complications and lower mortality rates, particularly among high‐risk patients undergoing PCI [1]. The current guidelines have endorsed TRA for invasive evaluations in patients with acute coronary syndrome (ACS), further solidifying its acceptance among interventional cardiologists [2].

Despite these advantages, TRA is not without risks, as it can lead to rare yet severe complications, including perforations in the subclavian vessels, brachiocephalic artery, internal mammary artery, and thyrocervical trunk. Such complications, although uncommon, carry potentially life‐threatening consequences and necessitate careful procedural execution. This report discusses a case of a perforation in the right internal mammary artery (RIMA) during TRA coronary angiography, highlighting its successful management. Furthermore, it reviews the broader spectrum of TRA‐related perforations and associated complications.

## 2 Case Presentation

**FIGURE 1:**
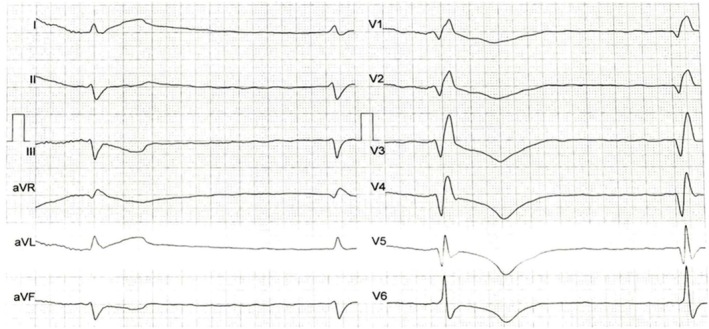
Initial ECG that showed AF with long‐duration pauses and a ventricular escape rhythm.

**FIGURE 2:**
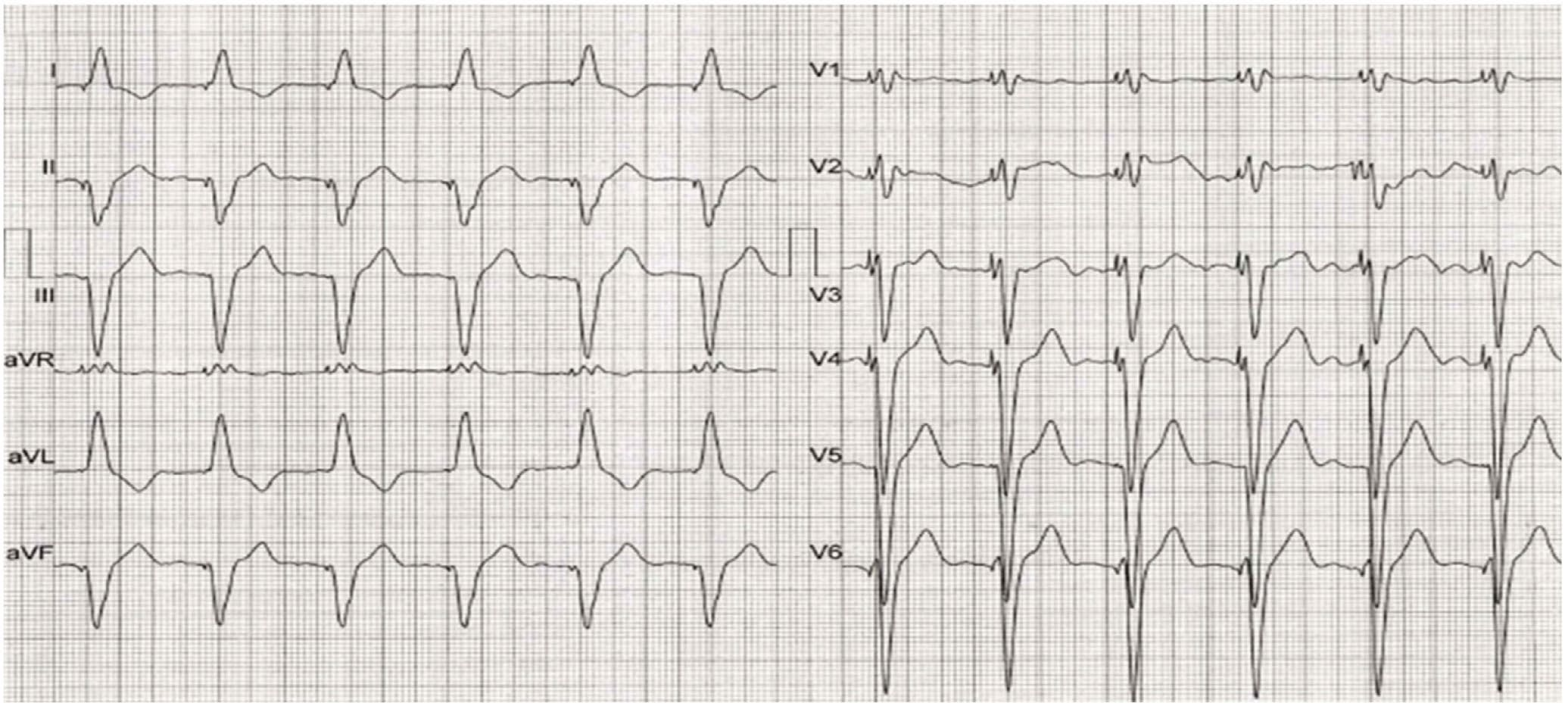
ECG obtained after temporary pacemaker insertion.

A 75‐year‐old woman presented to the emergency department with a chief complaint of weakness. Her medical history was unremarkable. Initial ECG showed atrial fibrillation (AF) with long pauses and a ventricular escape rhythm (Figure 1). Given her clinical condition and ECG findings, a temporary pacemaker was successfully inserted via the right femoral vein without complications, followed by a repeat ECG (Figure 2). Troponin level was within the normal range.

After stabilization, echocardiography revealed a normal left ventricular (LV) size with severe systolic dysfunction (LVEF: 20%), a smoky pattern in the LV cavity without evidence of clot, grade III diastolic dysfunction, normal right ventricle (RV) size with moderate systolic dysfunction, and biatrial enlargement without significant valvular abnormality.

Myocardial perfusion imaging (MPI) and viability assessment were subsequently performed, demonstrating a nontransmural myocardial infarction (with sufficient viable tissue) in the apical and mid‐to‐basal segments of the anterior wall. The findings also showed severely reduced global function (LVEF: 22%), dilated LV volumes (EDV = 142 mL; ESV = 111 mL), akinesia of the septum, and hypokinesia of the apex, anterior, and inferior walls.

**FIGURE 3:**
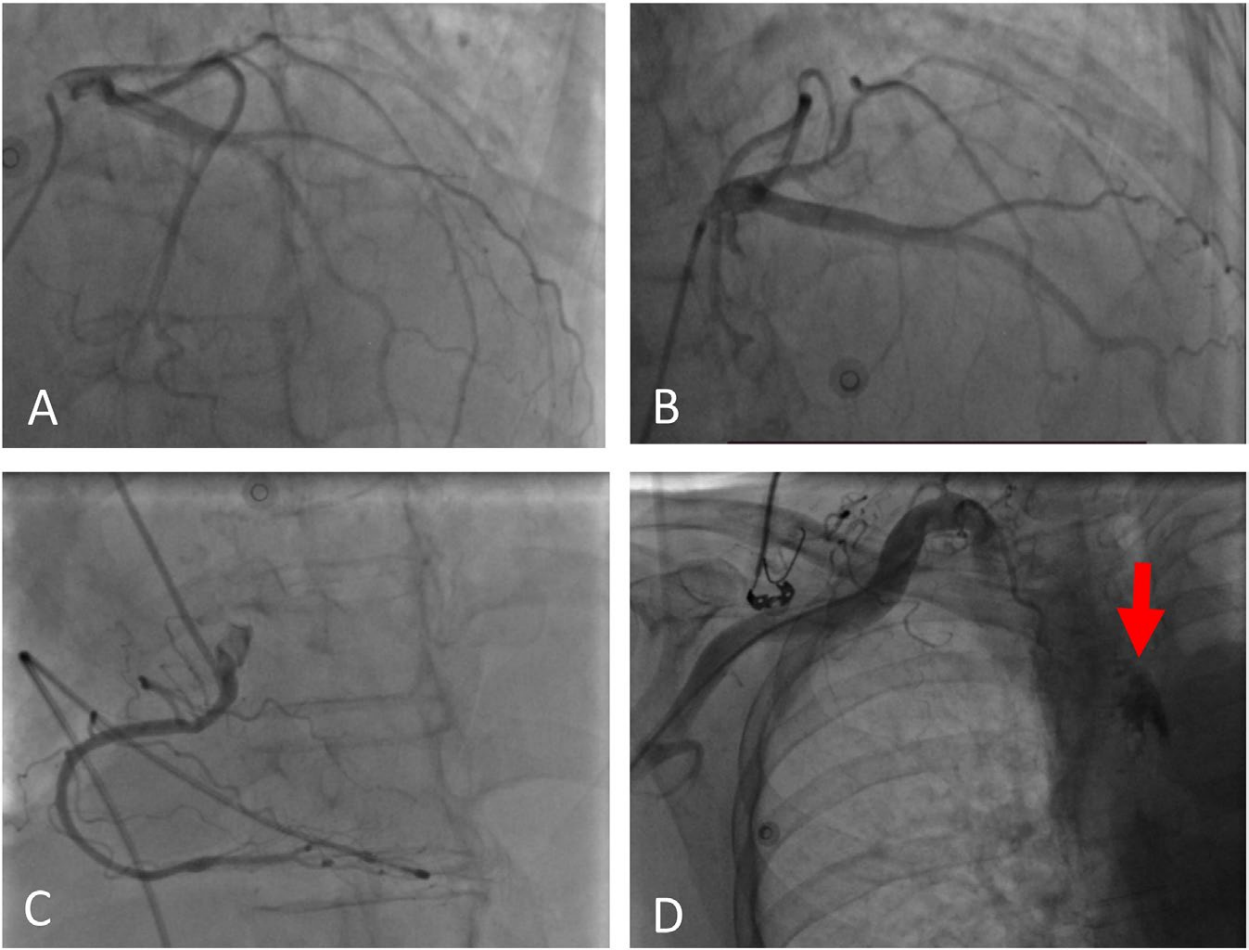
(A and B) Left coronary angiogram. (C) Right coronary angiogram. (D) Perforation of the side branch of RIMA shown in nonselective injection of the subclavian artery.

Given these findings, we decided to perform coronary angiography. The procedure was performed via the right radial artery (RRA), revealing normal coronary beds without lesions. However, the hydrophilic wire unintentionally passed through the RIMA during the procedure. To rule out perforation, we injected nonselective contrast into the aorta and the subclavian artery, showing tissue staining suggestive of severe spasm without an apparent perforation jet (Figure 3). The patient was initially managed conservatively with close observation.

In the cardiac care unit (CCU), the patient was closely monitored with serial hemoglobin checks and clinical assessments. On the second day, laboratory results indicated a significant drop in hemoglobin, from 14.6 g/dL to 9.7 g/dL.

### 2.1 Differential Diagnosis

A drop in hemoglobin after any interventional procedure should prompt emergent evaluation of potential procedure site complications. Moreover, internal bleeding, such as gastrointestinal (GI) bleeding due to periprocedural anticoagulation, should be suspected.

### 2.2 Investigation

**FIGURE 4:**
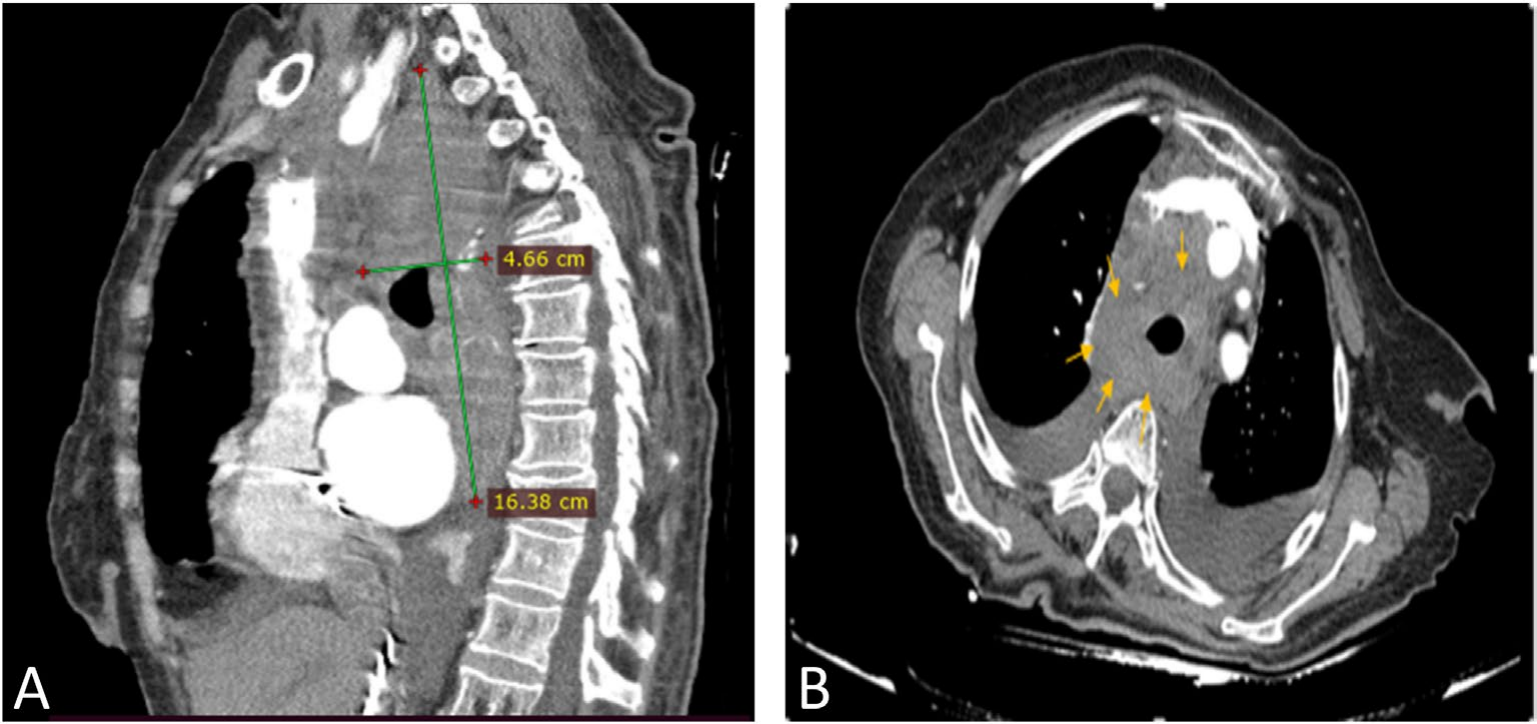
Significant hematoma in the mediastinum around the trachea and esophagus. (A) Sagittal view. (B) Axial view.

Emergent CT angiography of the thorax showed a significant hematoma in the mediastinum around the trachea and esophagus (Figure 4).

### 2.3 Management

**FIGURE 5:**
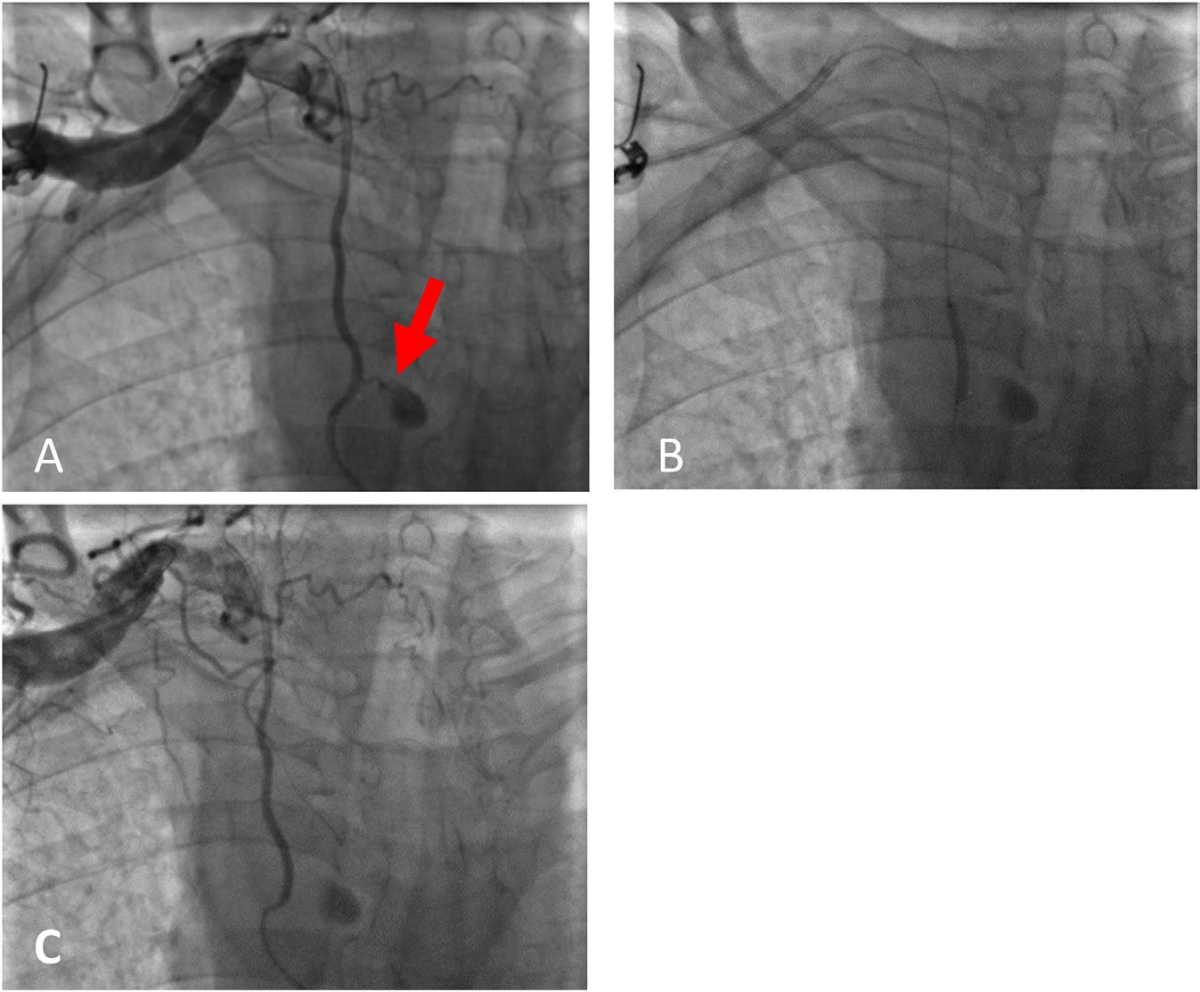
(A) Significant dye extravasation in the mediastinum. (B) Deployment of the covered stent. (C) Poststenting injection, no new extravasation was seen.

The patient was emergently transferred to the catheterization laboratory to address the potential bleeding. Using the TRA, a catheter was passed to the subclavian artery. Subclavian artery injection showed significant dye extravasation into the mediastinum, confirming perforation of the RIMA. A guiding catheter was introduced, and the RIMA was successfully wired with a Galeo Pro 0.014‐inch guidewire. Balloon tamponade was performed using a Filao NC 2.5 × 15 mm balloon. Despite three prolonged balloon inflations, the perforation remained unsealed. As a result, stenting with a covered stent was planned. A 2.5 × 21 mm stent graft (Begraft) was deployed. Postdeployment angiography revealed no further dye extravasation, indicating successful sealing of the perforation. The procedure was terminated with a good final result (Figure 5).

### 2.4 Follow‐Up

She was monitored closely in the CCU for 24 h, during which her hemoglobin level stabilized, and her clinical condition improved significantly. A permanent cardiac resynchronization therapy (CRT) device was also inserted during the hospital course. Then she was subsequently discharged in stable condition. No complications occurred during the 6‐month follow‐up after discharge.

## 3 Discussion

This report describes a rare complication of transradial coronary angiography: right internal mammary artery (RIMA) perforation resulting in a large mediastinal hematoma. Only two previous cases of RIMA perforation during TRA have been published—one managed with embolization during a neurovascular procedure [3] and another treated with a handmade stent graft [3]. Our case is distinctive because the perforation initially appeared as a severe spasm without overt extravasation, which delayed its recognition, and was ultimately managed with a commercially available covered stent after balloon tamponade failed.

The key lesson from this case is that an apparently benign wire‐induced spasm may conceal an evolving perforation. In our patient, close monitoring and timely imaging were crucial, as hemoglobin decline provided the first clear evidence of ongoing bleeding despite reassuring early angiography. This emphasizes the need for vigilance and a low threshold for repeat imaging when clinical deterioration occurs after suspected vessel trauma.

From a therapeutic standpoint, covered stenting proved to be a life‐saving intervention. While embolization has been successfully used in some reports, it is not always feasible, particularly when the injured vessel contributes to myocardial perfusion. Our case demonstrates that stent‐graft implantation can be an effective and durable solution for sealing RIMA perforations, adding to the limited experience available in the literature.

Taken together, this case highlights three clinically relevant messages: (1) RIMA perforation is a rare but serious TRA complication; (2) initial angiography may underestimate the severity of injury, underscoring the importance of vigilant follow‐up; and (3) covered stent deployment represents a viable treatment option when balloon tamponade fails.

This is a case report of RIMA perforation during CAG through TRA, which was successfully managed with stenting. Common complications of the TRA angiography include puncture site hematoma, perforation, dissection, and radial artery occlusion. Cerebrovascular events are also rare but documented. There are reports of major central arterial complications, such as subclavian artery perforation, brachiocephalic trunk dissection, and vertebral artery perforation, which have been comprehensively reviewed by Wang et al. [4]. Only one previous case report had reported RIMA perforation as the sequence of a neurovascular procedure [5] and a coronary angiogram [3] with TRA. Abecassis et al. in 2021 reported an 87‐year‐old man with sudden right eye blurred vision who had undergone a neurovascular procedure. He developed a growing hematoma in the right pectoral area after the procedure due to RIMA perforation. The case was successfully managed with embolization, as the RIMA did not supply myocardial or transplanted tissue [5]. Tatli et al. in 2014 presented a 72‐year‐old female patient with a right internal mammary artery perforation following transradial CAG, which was managed with the implantation of a handmade stent graft [3].

In our case, CAG was performed by an experienced interventional cardiologist. However, due to severe tortuosity in the brachial and subclavian arteries, it became necessary to use a hydrophilic 0.035‐inch guiding wire to pass through. Passing a hydrophilic wire, which has a potent risk of dissection and perforation, unintentionally caused a perforation of the RIMA. Initially, RIMA angiography showed minimal dye extravasation, prompting conservative management. However, the patient's progressive drop in hemoglobin levels and clinical decompensation necessitated intervention. Similar to other forms of coronary or peripheral artery perforation, balloon tamponade was attempted, but it was not successful. Consequently, a covered stent was deployed to seal the small bleeding branch of the RIMA.

Clinical evidence indicates that complications associated with TRA are generally less frequent and more severe than those seen with the transfemoral approach. Most TRA‐related complications are confined to areas below the elbow [6]. However, in rare cases, TRA can result in severe outcomes, such as subclavian artery entrapment or perforation of the cephalobrachial, thyroid, carotid, or internal mammary arteries [7]. Notably, Luo et al. identified that thoracic hematomas are predominantly associated with hydrophilic guidewires during transradial cardiac catheterization [8]. These guidewires have the potential to cause perforations or hematomas in any branch of the arteries they traverse.

A review of reported cases reveals that internal mammary artery perforations frequently occur during PCI procedures involving the left internal mammary artery (LIMA) graft, which falls outside the scope of this case report. However, there are documented cases of RIMA branch ruptures during TRA. For example, one case involved a trajectory misinterpretation during a neurovascular procedure, leading to RIMA perforation and subsequent hematoma, which required coil and glue embolization for resolution [5]. Other examples of rare TRA‐related deep vascular complications have been documented. Choi et al. described a mediastinal hematoma caused by a hydrophilic wire during PCI, which necessitated interventional occlusion and hematoma aspiration [9]. Similarly, Sharma et al. reported a right axillary artery branch perforation leading to a large chest wall hematoma, successfully treated with autologous clot‐blocking [10].

Prompt recognition and management are critical for perforated vessels, as delayed intervention can worsen patient outcomes. While larger arterial perforations often necessitate covered stents, smaller branch perforations are effectively managed with embolization using materials like spring coils, gelatin sponges, or autologous clot blocks [11]. For example, Abecassis and Choi utilized a combination of spring coils and gelatin sponges to prevent hematoma expansion in their respective cases [5, 9]. TRA‐related thoracic hematomas can exert significant pressure on adjacent organs, occasionally requiring surgical intervention. For instance, in the case reported by Choi et al., mediastinoscopic aspiration was necessary to alleviate suffocation caused by a hematoma [9]. In this case, lung function deterioration prompted surgical removal of the hematoma alongside valve replacement and ascending aorta replacement surgeries. A key feature of this case is that the initial RIMA angiography did not reveal an obvious perforation, underscoring the importance of close observation, even when early imaging appears reassuring.

## 4 Conclusion

Although TRA generally reduces access‐related complications compared with transfemoral angiography, rare but catastrophic vascular injuries such as RIMA perforation can still occur. The novelty of our case lies in (1) the delayed presentation of perforation despite initially reassuring imaging, and (2) the successful use of a covered stent to seal the RIMA. This report underscores the need for close clinical monitoring after suspected wire‐induced vessel trauma. It demonstrates that covered stenting provides an effective therapeutic strategy when conservative measures or balloon tamponade fail. Awareness, early detection, and tailored management of such complications are vital to improving patient outcomes.

## Author Contributions

**Seyed Alireza Mirhosseini:** investigation, visualization, writing – original draft, writing – review and editing. **Mahsa Borjzadehgashtaseb:** investigation. **Mohammad Mohammadi:** investigation. **Soorena Khorshidi:** investigation, writing – original draft. **Foad Amanollahi:** investigation. **Alisina Mirzaei:** investigation, writing – original draft. **Armin Attar:** conceptualization, supervision.

## Funding

The authors have nothing to report.

## Ethics Statement

The authors have nothing to report.

## Consent

Written informed consent was obtained from the patient for this study.

## Conflicts of Interest

The authors declare no conflicts of interest.

## Data Availability

The authors have nothing to report.
